# Plasma big endothelin-1 predicts new-onset atrial fibrillation after surgical septal myectomy in patients with hypertrophic cardiomyopathy

**DOI:** 10.1186/s12872-019-1085-4

**Published:** 2019-05-22

**Authors:** Changpeng Song, Shengwei Wang, Ying Guo, Xinxin Zheng, Jie Lu, Xiaonan Fang, Shuiyun Wang, Xiaohong Huang

**Affiliations:** 1 Department of Special Medical Treatment Center, Fuwai Hospital, National Center for Cardiovascular Diseases, Chinese Academy of Medical Science, Peking Union Medical College, 167 BeiLiShi Road, Xichen District, Beijing, 100037 People’s Republic of China; 2 Department of Cardiovascular Surgery, Fuwai Hospital, National Center for Cardiovascular Diseases, Chinese Academy of Medical Science, Peking Union Medical College, 167 BeiLiShi Road, Xichen District, Beijing, 100037 People’s Republic of China

**Keywords:** Big endothelin-1, Hypertrophic cardiomyopathy, Postoperative atrial fibrillation, Surgery

## Abstract

**Background:**

Postoperative atrial fibrillation (POAF) is a common complication in patients with obstructive hypertrophic cardiomyopathy (HOCM) who undergo surgical myectomy. POAF is associated with poor outcome. The role of plasma big endothelin-1 level in predicting atrial fibrillation after surgical septal myectomy in HOCM patients has not well been studied.

**Methods:**

A total of 118 patients with HOCM who underwent surgical septal myectomy were recruited in this study. Plasma big endothelin-1 level was measured. The heart rhythm was continuously monitored during hospital stay. Preoperative, intraoperative, and postoperative variables were collected.

**Results:**

POAF developed among 26 of the 118 patients (22%) in this study. Compared with those without POAF, patients with POAF were significantly older (53.5 ± 10.6 vs. 47.3 ± 13.6 years, *P* = 0.033), more likely to undergo mitral valve surgery (38.5% vs. 18.5%, *P* = 0.032), and had higher plasma big endothelin-1 levels (0.41 ± 0.19 vs. 0.27 ± 0.14 pmol/l, *P* = 0.001), longer hospital stay (9.1 ± 3.7 vs. 7.5 ± 2.8 days, *P* = 0.022), larger preoperative left atria (48.0 ± 5.2 vs. 44.1 ± 5.9 mm; *P* = 0.003). In the receiver operating characteristic curve analysis, the area under the curve for big endothelin-1 was 0.734 (95% CI, 0.634 to 0.834, P<0.001). In multivariate logistic regression analysis, preoperative big endothelin-1 level (OR 100.7, 95%CI: 5.0–2020.0, *P* = 0.003) and left atrial diameter (OR 1.106, 95%CI: 1.015–1.205, *P* = 0.022) were independent predictors of POAF.

**Conclusion:**

Elevated preoperative plasma big endothelin-1 level is an independent predictor of POAF in HOCM patients undergoing surgical septal myectomy.

## Background

Postoperative atrial fibrillation (POAF) is a common complication in patients with obstructive hypertrophic cardiomyopathy (HOCM) who undergo surgical myectomy, occurring in 17–20% of patients [1, 2] Although POAF may present as a benign condition, it sometimes has an adverse influence on patients as it contributes to increased complications, mortality, and duration of hospital stay [2]. Thereby, it is important to investigate valuable markers to identify those who are at high risk of POAF and provide AF prophylaxis and intensive care perioperative procedure.

Endothelin-1, one of the most potent vasoconstrictors, is released in response to various stimuli, including cardiac ischemia, increased wall stress and fulfilling pressure [3]. Endothelin-1 has been reported to be associated with left atrial dilatation, fibrosis and atrial fibrillation [4]. Furthermore, it has also been used to predict the occurrence of POAF after mitral valve surgery [5] and recurrence of AF after catheter ablation [6]. Big endothelin-1, the precursor hormone of endothelin-1, has also been shown to be associated with atrial fibrillation and many other cardiac diseases. In HCM, Tang, et al. demonstrated that elevated plasma big endothelin-1 level is an independent predictor of long-term survival [7].

Nevertheless, the role of plasma big endothelin-1 level in predicting POAF after surgical septal myectomy in HOCM patients has not been well investigated. This present study aimed to assess the impact of preoperative plasma big endothelin-1 level on new-onset AF after surgical septal myectomy.

## Methods

Patients who underwent surgical septal myectomy were assessed at Fuwai Hospital, Chinese Academy of Medical Sciences, between January 2013 and March 2018. The diagnosis of HCM was based on the presence of myocardial hypertrophy (maximum wall thickness ≥ 15 mm) in the absence of any other cardiac or systemic cause resulting in the cardiac hypertrophy. Surgical septal myectomy was introduce to those with drug-refractory symptoms and the maximum left ventricular outflow tract (LVOT) gradient or midventricular gradient ≥50 mmHg at rest or with physiologic provocation, and without severe liver or renal failure. The major exclusion criteria were previous history of atrial arrhythmia (paroxysmal or permanent AF, AF surgical or catheter ablation, atrial flutter, and other type atrial tachycardia) and a history of implanted cardiac pacemaker. Demographic information, clinical data and medications were collected. The study was approved by the Ethics Committees of Fuwai Hospital, Chinese Academy of Medical Sciences. All patients consented to provide clinical data for research purposes.

All patients underwent preoperative, postoperative echocardiograms including 2-dimensional and Doppler type. Basal subaortic and midventricular gradients were measured with continuous Doppler in the apical 3-chamber view. We assessed quantitatively left ventricular (LV) end-diastolic diameter, LV ejection fraction, LV wall thickness, and left atrial diameter following recommendations of the American Society of Echocardiography [8]. Resting LVOT obstruction was documented when a peak gradient ≥30 mmHg in a normal condition was identified by Doppler [9]. Mitral regurgitation was classified as mild, moderate and severe. Pulmonary hypertension was defined as a pulmonary artery systolic pressure ≥ 35 mmHg.

As described previously [10], patients underwent a transaortic extended Morrow procedure. The hypertrophic ventricular septum leading to LVOT obstruction or systolic anterior motion of the mitral valve was resected. If a postoperative LVOT gradient detected by intraoperative transoesophageal echocardiography > 30 mmHg after weaning from cardiopulmonary bypass, reoperation was required. Concomitant surgeries, including coronary artery bypass graft, myocardial unroofing, and valve surgery, were performed based on expert consensus among the experienced cardiac surgeons. Perioperative and postoperative data were recorded.

All patients underwent continuous cardiac monitoring using a 5-lead telemetry strip during postoperative hospital stay. A standard 12-lead electrocardiogram was routinely obtained and checked daily. POAF was defined as an episode of AF lasting for more than 5 min or requiring antiarrhythmic therapy or electrical cardioversion. When necessary to confirm the diagnosis of POAF, additional 12-lead electrocardiograms and electrocardiographic Holter monitoring were performed.

Venous blood samples were drawn before operation. Plasma was isolated after centrifugation. Plasma big endothelin-1 concentrations were determined by Big Endothelin-1 ELISA Kit (Biomedica Medizinprodukte GmbH & Co KG, Austria), after the manufacturer introductions.

Continuous variables were presented as mean ± standard deviation. Categorical measures were presented as number (percentage). Comparison of continuous variables between groups was performed by Student^,^ s t test (normally distributed) or Mann-Whitney U test (non-normally distributed), accordingly. Comparison of categorical data was performed using the chi-square or Fisher’s exact tests. The cut-off value for big ENDOTHELIN-1 was determined by area under the receiver-operator characteristic (ROC) curve. Both univariate and multivariate logistic regression analyses were conducted to determine the predictors of POAF. Nonparametric variables were log transformed. Variables with a *p* value < 0.1 by univariate analysis were included in a multivariable logistic regression model. A *P*-value < 0.05 (two-sided) was considered statistically significant. Statistical analyses were performed with SPSS version 21.0 (IBM Corp, Armonk, NY).

## Results

Preoperative and postoperative clinical characteristics were shown in Table 1. Of all the patients, the mean age was 48.7 ± 13.2 years (63.6% male). Median big endothelin-1 for the entire study population was 0.25 (interquartile range [IQR]: 0.18 to 0.41) pmol/l. POAF developed in 26 of the 118 patients (22%) who underwent surgical septal myectomy during postoperative hospital stay. Compared with those without POAF, patients with POAF were significantly older (53.5 ± 10.6 vs. 47.3 ± 13.6 years, *P* = 0.033), more likely to undergo mitral valve surgery (38.5% vs. 18.5%, *P* = 0.032), and had higher plasma big endothelin-1 levels (0.41 ± 0.19 vs. 0.27 ± 0.14 pmol/l, *P* = 0.001) and longer postoperative hospital stay (9.1 ± 3.7 vs. 7.5 ± 2.8 days, *P* = 0.022). Preoperative and postoperative echocardiographic data were summarized in Table 2. Patients who had POAF had larger preoperative left atria (48.0 ± 5.2 vs. 44.1 ± 5.9 mm; *P* = 0.003) and postoperative left atria (40.2 ± 4.8 vs. 36.9 ± 5.3 mm; *P* = 0.004). No significant associations were identified between POAF development and other echocardiographic variables.

**Table 1:** Pre- and post-operative clinical variables of patients

Variables	Whole cohort (*n* = 118)	POAF (*n* = 26)	No POAF (*n* = 92)	*P*-value
Preoperative data				
Age (years)	48.7 ± 13.2	53.5 ± 10.6	47.3 ± 13.6	0.033
Male (%)	75 (63.6)	15 (57.7)	60 (65.2)	0.48
Hypertension (%)	27 (22.9)	7 (26.9)	20 (21.7)	0.58
Diabetes mellitus (%)	4 (3.4)	1 (3.8)	3 (3.3)	1.00
CAD (%)	14 (11.9)	2 (7.6)	12 (13.0)	0.73
Body mass index (kg/m^2^)	25.0 ± 3.3	25.9 ± 3.1	24.8 ± 3.3	0.14
Big endothelin-1 (pmol/l)	0.30 ± 0.16	0.41 ± 0.19	0.27 ± 0.14	0.001
Medication				
Calcium channel blockers (%)	11 (9.3)	2 (7.6)	9 (9.9)	1.000
Beta-blockers (%)	115 (97.5)	26 (100)	89 (96.7)	1.000
Concomitant operative procedures				
CABG or myocardial unroofing (%)	23 (19.5)	3 (11.5)	20 (21.7)	0.246
Aortic valve replacement or repair (%)	2 (1.7)	0 (0)	2 (2.2)	1.000
Mitral valve replacement or repair (%)	27 (22.9)	10 (38.5)	17 (18.5)	0.032
Tricuspid valve replacement or repair (%)	11 (9.3)	5 (19.2)	6 (6.5)	0.063
Postoperative data				
Mechanical ventilation time (hours)	21.6 ± 17.0	26.2 ± 28.2	20.3 ± 12.0	0.31
Aortic clamp time (minutes)	73.1 ± 29.9	75.0 ± 25.0	72.6 ± 31.3	0.71
Post-operative hospital stay (days)	7.9 ± 3.1	9.1 ± 3.7	7.5 ± 2.8	0.022

*CAD* coronary artery disease, *CABG* Coronary Artery Bypass Grafting, *POAF* postoperative atrial fibrillation

**Table 2:** Pre- and post-operative echocardiographic variables of patients

Variables	POAF (*n* = 26)	No POAF (*n* = 92)	*P*-value
Preoperative data			
Maximum wall thickness (mm)	21.9 ± 4.6	22.1 ± 4.3	0.81
Left atrial diameter (mm)	48.0 ± 5.2	44.1 ± 5.9	0.003
Left ventricular end-diastolic diameter (mm)	43.2 ± 4.4	43.1 ± 4.3	0.96
Left ventricular ejection fraction (%)	71.6 ± 5.0	70.7 ± 5.7	0.48
LVOT obstruction at rest (%)	22 (84.6)	83 (90.2)	0.42
Moderate or severe MR (%)	19 (73.1)	60 (67.8)	0.45
Pulmonary hypertension (%)	3 (11.5)	3 (3.3)	0.12
Postoperative data			
Left atrial diameter (mm)	40.2 ± 4.8	36.9 ± 5.3	0.004
Left ventricular end-diastolic diameter (mm)	45.7 ± 3.8	45.1 ± 5.1	0.64
Left ventricular ejection fraction (%)	60.9 ± 5.4	63.0 ± 5.1	0.074
Residual LVOT gradients (mmHg)	13.6 ± 4.8	13.8 ± 7.3	0.88

*LVOT* left ventricular outflow tract, *MR* mitral regurgitation

**Fig. 1:**
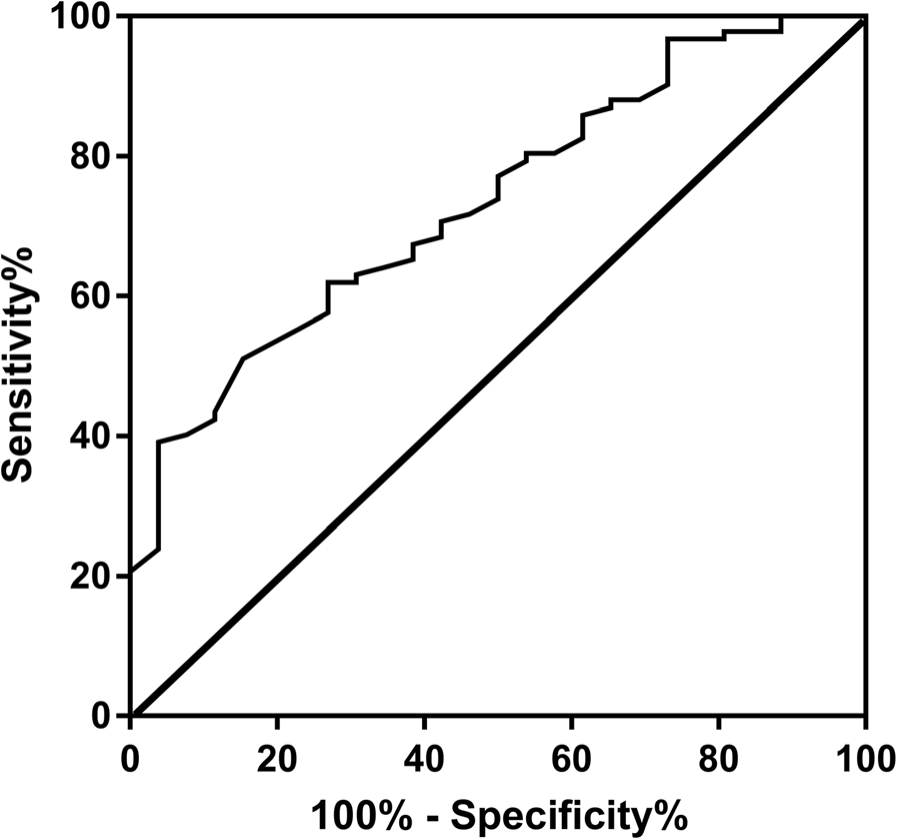
Receiver operating characteristic curves of plasma big endothelin-1 level for predicting AF after surgical septal myectomy. The optimal cut-off point was 0.235 pmol/l predicted AF after myectomy with sensitivity and specificity of 51 and 84% (area under the curve 0.734, 95% CI: 0.634 to 0.834, *P* < 0.001)

We conducted ROC curve analysis to assess the ability of big endothelin-1 levels to identify patients with and without POAF (Fig. 1). The area under the curve for big endothelin-1 was 0.734 (95% CI: 0.634 to 0.834, P<0.001). A big endothelin-1 level of 0.235 pmol/l was the optimal cut-off point to predict AF after surgical septal myectomy, with a sensitivity 0.51 and specificity of 0.84. Then, patients were divided into 2 groups: high big endothelin-1 group (*n* = 67) and low big endothelin-1 group (*n* = 61). Patients in high big endothelin-1 group were significantly older (51.0 ± 12.3 vs. 45.7 ± 13.9 years, *P* = 0.031) and more likely to have left atrial dilatation (46.0 ± 5.4 vs. 43.5 ± 6.4 mm, *P* = 0.019), moderate or severe mitral regurgitation (74.6% vs. 70.5%, *P* = 0.042) and pulmonary hypertension (8.8% vs. 0%, *P* = 0.036). More POAF occurred in patients with big endothelin-1>0.235 pmol/l (32.4% vs. 6.6%, *P* = 0.001).(Details shown in Table 3).

**Table 3:** Comparison of clinical variables between high and low big endothelin-1 groups

Characteristics	High big ET-1 group (*n* = 67)	Low big ET-1 group (*n* = 61)	*P*-value
Age (year)s	51.0 ± 12.3	45.7 ± 13.9	0.031
Male (%)	41 (61.2)	34 (55.8)	0.54
Body mass index (kg/m^2^)	25.5 ± 3.2	24.4 ± 3.4	0.06
Hypertension (%)	16 (23.9)	11 (18.0)	0.77
Left atrial diameter (mm)	46.0 ± 5.4	43.5 ± 6.4	0.019
LV end-diastolic diameter (mm)	43.1 ± 4.2	43.1 ± 4.5	0.97
LV ejection fraction (%)	70.9 ± 5.7	71.0 ± 5.5	0.92
Maximum wall thickness (mm)	21.7 ± 4.0	22.6 ± 4.7	0.24
LVOT obstruction at rest (%)	62 (92.5)	43 (70.5)	0.24
Moderate or severe MR (%)	50 (74.6)	29 (47.5)	0.042
Pulmonary hypertension (%)	6 (9.0)	0 (0)	0.036
POAF (%)	22 (32.8)	4 (6.6)	0.001

*ET-1* endothelin-1, *LV* left ventricular, *LVOT* left ventricular outflow tract

The univariate and multivariate logistic regression analyses were conducted to evaluate the preoperative predictors of POAF. In the univariate analysis, preoperative variables including age (odds ratio (OR): 1.043, 95% confidence interval (CI)): 1.002–1.084, *P* = 0.038), left atrial diameter (OR: 1.123, 95% CI: 1.036–1.217, *P* = 0.005), and big endothelin-1 (OR: 177.7, 95% CI: 10.5–3007, *P* < 0.001) were identified as the risk factors of POAF. In the multivariate analysis, preoperative big endothelin-1 level (OR 100.7, 95%CI: 5.0–2020.0, *P* = 0.003) and left atrial diameter (OR 1.106, 95%CI: 1.015–1.205, *P* = 0.022) were independent predictors of AF. (Details shown in Table 4).

**Table 4:** Logistic analysis for predictors of postoperative atrial fibrillation

Characteristics	OR	95% CI	*P*-value
Univariate Logistic regression analysis			
Age	1.043	1.002–1.084	0.038
Male	0.727	0.299–1.768	0.482
Body mass index	1.109	0.966–1.273	0.142
Hypertension	1.326	0.489–3.599	0.579
Left atrial diameter	1.123	1.036–1.217	0.005
Left ventricular end-diastolic diameter	1.002	0.905–1.110	0.962
Left ventricular ejection fraction	1.029	0.951–1.114	0.476
Maximum wall thickness	0.987	0.892–1.092	0.806
LVOT obstruction at rest	0.596	0.168–2.120	0.424
Moderate or severe mitral regurgitation	1.448	0.550–3.807	0.453
Pulmonary hypertension	3.870	0.732–20.447	0.111
Big ET-1	177.7	10.5–3007	< 0.001
Multivariate Logistic regression analysis^a^			
Age	1.034	0.991–1.080	0.126
Left atrial diameter	1.106	1.015–1.205	0.022
Big ET-1	100.7	5.0–2020.0	0.003

*CI* confidence interval, *ET-1* endothelin-1, *LVOT* left ventricular outflow tract, *OR* odds ratio

^a^Age, left atrial diameter, and high big ET-1 versus low big ET-1 were included in the multivariate logistic regression analysis

## Discussion

Data on the risk factors of POAF in patients who underwent surgical septal myectomy is limited. The present study has been the first study to investigate the association between big endothelin-1 and new-onset AF after surgical septal myectomy. Our data indicates that elevated plasma big endothelin-1 level is an independent predictor of POAF among these patients. In addition, increased left atrial diameter also can predict new-onset AF after surgical septal myectomy.

AF occurred among 22% of our patients during postoperative hospital stay. This rate is similar to that reported previously [1, 2]. In line with evidence from previous reports [1], patients who developed POAF have a longer postoperative hospital stay in this study. As few adverse events occurred, we have not assessed the POAF with perioperative adverse events.

Left atrial size is an indirect indicator of left ventricular fulfilling pressure, which is often increased in patients with HCM, especially in those with LVOT obstruction and moderate or severe mitral regurgitation caused by systolic anterior motion of the mitral valve [11]. Previous studies demonstrated the association of enlarged left atria with AF in patients undergoing surgical septal myectomy [12]. In this study, we demonstrated the associations of POAF with pre- and post-operative left atrial size. This finding indicated that the structural remodeling of left atria influenced the occurrence of AF after surgical septal myectomy.

Furthermore, we also found that plasma big endothelin-1 level was an independent predictor of POAF. Endothelin-1, one of the most potent vasoconstrictors, is synthesized and secreted mostly in endothelial cells. Increased plasma endothelin-1 levels have been reported to be associated with advanced age [5]. In this study, our data was consistent with this previous finding. In addition, plasma endothelin-1 levels were reported to be associated with increased atrial pressure and atrial size [13]. The relevance of atrial endothelin-1 level to atrial size was also demonstrated by Mayyas, et al. [4] In HCM, our data, combined with results reported by Tang, et al. [7], showed that plasma big endothelin-1 level was positively correlated with left atrial diameter. In experimental study, overexpressed endothelin-1 significantly facilitated atrial hypertrophy and dilatation [14]. Left atrial dilatation is one of the most frequent causes of AF. The impact of endothelin-1 on atrial structure partially explained the association between endothelin-1 and POAF.

Atrial fibrosis, one common pathological remodeling of left atria, contributes to the development of AF [15]. Atrial fibrosis builds a substrate for AF, which could perturb anisotropic conduction, decrease conduction velocities, and lead to reentry circuits [1]. Fibrosis may be another link between AF and endothelin-1. Mayyas, et al. have demonstrated the association of endothelin-1 with platelet derived growth factor (PDGF) and connective tissue growth factor (CTGF) [4]. Both of PDGF [16] and CTGF [17] signaling pathways have been reported to be involved in the cardiac fibrosis development. Moreover, the relationship of endothelin-1 and collagen accumulation was also identified by the analysis of Masson’s trichrome stained sections [4]. Therefore, we conclude that endothelin-1 could be an important modulator of cardiac fibrosis. In HCM, plasma big endothelin-1 has been reported to be in positive correlation with late gadolinium enhancement on magnetic resonance imaging [7]. The important role of endothelin-1 in fibrosis development could be another mechanism of POAF since atrial fibrosis is another prominent cause of AF.

In the addition to promoting atrial dilatation and cardiac fibrosis, endothelin-1 could cause POAF through many other pathways. In mammalian models, endothelin-1 suppresses electrical excitability of the heart by inhibiting the L-type calcium current and activating the muscarinic potassium current [18]. Besides, plasma endothelin-1 levels have been reported to be positively correlated with high-sensitivity C-reactive protein which is a marker of inflammation [19]. Inflammatory response was also associated with AF and the development of new-onset AF after cardiac surgery [20].

Although the exact role of endothelin system in the occurrence of POAF, we found that elevated plasma big endothelin-1 level was the independent predictor of POAF in HCM patients who underwent surgical septal myectomy. Preoperative measurement of big endothelin-1 may help in the risk stratification of POAF for patients with HOCM who desired to undergo surgical septal myectomy. Furthermore, more studies might be needed to investigate the role of medical strategies protecting endothelin-1 levels in POAF prevention in the future.

## Limitations

Our study has several limitations. First, the study was conducted retrospectively in a single tertiary center. Therefore, this work might have selecting bias. Second, the assessment of related biomarkers including brain natriuretic peptide is very meaningful, but we cannot get all the information of the additional biomarkers. Third, the number of our patients was limited. This might partly explain why some factors which have been illustrated to be significantly related to AF were not statistically significant in this study. Therefore, a prospective, multicenter, and large sample size study are needed.

## Conclusions

An elevated preoperative plasma big endothelin-1 level is an independent predictor of POAF in HOCM patients undergoing surgical septal myectomy. Our data provide additional predictors of POAF in patients undergo septal myectomy.

## Abbreviations

AF: Atrial fibrillation
HCM: Hypertrophic cardiomyopathy
HOCM: Obstructive hypertrophic cardiomyopathy
LV: Left ventricular
LVOT: Left ventricular outflow tract
POAF: Postoperative atrial fibrillation
